# Identifying a prognostic signature for clear cell renal cell carcinoma: the convergence of single-cell and bulk sequencing with machine learning

**DOI:** 10.3389/fcell.2025.1560095

**Published:** 2025-06-04

**Authors:** Yude Hong, Xiao Hu, Libo Chen, Mingyong Li, Mingxiao Zhang, Weiming Deng

**Affiliations:** ^1^ Department of Urology, The Second Affiliated Hospital, Hengyang Medical School, University of South China, Hengyang, China; ^2^ Department of Urology, Affiliated Beijing Chaoyang Hospital of Capital Medical University, Beijing, China; ^3^ Department of Urology, The First Affiliated Hospital, Hengyang Medical School, University of South China, Hengyang, China; ^4^ Department of Urology, China-Japan Friendship Hospital, Beijing, China

**Keywords:** clear cell renal cell carcinoma, immunotherapy, machine learning, prognostic signature, single-cell RNA-seq

## Abstract

**Background:**

Clear cell renal cell carcinoma (ccRCC) is a highly aggressive renal cancer subtype and lacks highly precise individualized treatment options. Thus, we used a novel computational framework to construct a consensus machine learning-related signature (MLRS) to predict prognosis and screen patients effectively for immunotherapy.

**Methods:**

An integrative machine learning procedure involving 10 methods was used to contract MLRS. Various methods were used to evaluate immune cell infiltration and biological characteristics. Moreover, we explored the response to immunotherapy and drug sensitivity. Single-cell RNA sequencing analysis, qRT-PCR, and a CCK-8 assay were used to clarify the biological functions of the hub gene.

**Results:**

MLRS demonstrated outstanding performance in predicting prognosis compared with the other published signatures, and the high-MLRS group had a favorable outcome in four independent datasets. Furthermore, the low-MLRS group displayed a greater possibility of responding to immunotherapy and had a “hot” tumor immunophenotype. The high-MLRS group was characterized by a phenotype of immune suppression and was less likely to benefit from immunotherapy, while some small molecule inhibitors might serve as promising treatment options. Single-cell analysis revealed that MLRS was highly enriched in endothelial cells. We also identified DLL4/Notch and JAG/Notch signaling as the key ligand-receptor pairs in ccRCC. EMCN was downregulated in ccRCC, and further functional experiments demonstrated that EMCN knockdown inhibited cell proliferation.

**Conclusion:**

The MLRS can predict patient prognosis, may be utilized to screen potential populations that may benefit from immunotherapy, and predict potential drug targets, with broad significance for the clinical treatment of ccRCC.

## 1 Introduction

Kidney cancer is a prevalent malignancy with a rising incidence globally (Capitanio and Montorsi, 2016). According to GLOBOCAN 2020 (Global Cancer Statistics 2020), there were 431,288 diagnoses of kidney cancer and 179,368 fatalities attributed to the disease globally (Sung et al., 2021). Renal cell carcinoma (RCC) is the predominant form of kidney cancer in adults, with clear cell RCC (ccRCC) representing the most prevalent pathological subtype (Capitanio and Montorsi, 2016; Moch et al., 2022). Early diagnosis can result in a high probability of recovery; nevertheless, it is often challenging due to the insipid start and absence of conspicuous clinical signs (Capitanio et al., 2019). Although many novel therapeutic approaches have been proposed, surgical resection remains the most effective clinical treatment for ccRCC (Yin et al., 2023). Approximately 30% of patients with localised ccRCC ultimately develop metastatic recurrence during postoperative follow-up, resulting in a dismal prognosis (Kotecha et al., 2019; Quinlan et al., 2019; Taylor et al., 2023). Owing to resistance to standard chemotherapy and radiotherapy, therapies for ccRCC are still a significant challenge compared to those for other RCC subtypes (Ochocki et al., 2018). Recent tumor immunotherapy has been the focus in the clinical management of cancer and has shown favorable efficacy in certain cancer types (Powles et al., 2014; Gandhi et al., 2018; Huang and Zappasodi, 2022). While immunotherapies have become the standard of care for patients with advanced ccRCC, only a minority achieve significant clinical benefit (Darvin et al., 2018). Given the potential adverse effects and associated cost of immunotherapy, there is an urgent clinical need to identify biomarkers capable of facilitating effective outcome prediction to enable personalized cancer therapy in patients with ccRCC.

The clinical prognosis of ccRCC patients primarily correlates with traditional pathological indicators, including tumour grade and histological type (Mandal and Chan, 2016). Extensive intra- and inter-tumor heterogeneity may further confound the precision of pathological predictions (Huang et al., 2019). Integrating genomic abnormalities with clinical data demonstrated that several genetic mutations, such as chromatin-remodeling gene (*BAP1*) mutations, were independently correlated with poor survival in ccRCC patients (Linehan and Ricketts, 2019). Nonetheless, the true predictive significance of these indicators in clinical practice remains inadequately evaluated. Rapid advances in sequencing technologies and machine learning algorithms have allowed scientists to gain insight into these cancers at the molecular level (Ma et al., 2018). Comprehensive transcriptomic analysis has allowed for the identification of consensus molecular subtype classifications, which could contribute to addressing the difficulty in identifying the molecular heterogeneity of ccRCC.

In this study, we identified robust prognosis-related differentially expressed genes (DEGs) and constructed a consensus machine learning-related signature (MLRS) through a novel computational framework. The MLRS has great prognostic significance in multiple datasets and exhibits the robustness of its predictive value for immunotherapy and drug-based therapies. Ultimately, by integrating the single-cell RNA sequencing (scRNA-seq) data of clear cell renal cell carcinoma (ccRCC), we discovered that endomucin (*EMCN*) was linked to endothelial cell differentiation and suppressed the proliferation of ccRCC cells.

## 2 Materials and methods

### 2.1 Data collection

Initially, we acquired the multiomics data of ccRCC patients, including transcriptome expression, complete clinical information, and corresponding somatic mutation data, from the Cancer Genome Atlas (TCGA, https://portal.gdc.cancer.gov) database. The RNA-sequencing (RNA-seq) data were transformed to transcripts per kilobase million (TPM) values and subsequently normalized by log2 (TPM +1) transformation. We excluded patients with absent clinicopathological information and follow-up data, resulting in a final sample of 529 ccRCC samples from the TCGA dataset. The RNA-seq data and clinical data for the GSE22541 (Wuttig et al., 2012) were downloaded from the Gene Expression Omnibus (GEO, https://www.ncbi.nlm.nih.gov/geo/) database. The probes were converted into gene symbols utilizing the platform annotation file (Affymetrix Human Genome U133 Plus 2.0 Array). The RNA-seq and clinical data were acquired from the International Cancer Genome Consortium (ICGC, n = 91) and the E-MTAB-1980 (n = 101) databases.

### 2.2 Weighted gene co-expression network analysis (WGCNA)

WGCNA was performed to determine the ccRCC-specific network modules based on highly intercorrelated genes (Langfelder and Horvath, 2008). The gene co-expression network was constructed using a soft threshold power, allowing the network to approximate scale-free topology (scale-free fit signed *R* ^2^ > 0.9) in the TCGA dataset. The scale free topology model fit (signed *R* ^2^) measures how closely a gene co-expression network aligns with a scale-free topology, typically near 1 for a close match, while the soft threshold (Power) is used to weight correlations, emphasizing stronger connections between highly correlated gene pairs in the network construction (Langfelder and Horvath, 2008). We then generated a module-trait heatmap to illustrate the p values and correlation coefficients between the coexpression modules and ccRCC. In the present study, the most positively correlated and the most negatively correlated modules were termed the hub modules. The genes with module membership (MM) >0.8 and gene significance (GS) >0.5 were candidates for further analyses.

### 2.3 Construction of MLRS by integrative machine learning approaches

The “limma” R program was employed to identify differentially expressed genes (DEGs) in the TCGA dataset, comparing normal and ccRCC tissues with thresholds of |log2FoldChange| > 1.0 and p < 0.05. We then intersected the DEGs with the candidates obtained by WGCNA, which were then incorporated into the univariate analysis to identify potential prognostic genes. Finally, the genes with *p* < 0.05 were defined as prognostic-related genes (PRGs) and were then enrolled to construct a consensus MLRS with high generalizability.

To ensure model robustness and reproducibility, we systematically optimized hyperparameters for all 10 machine learning algorithms used in constructing the MLRS (Liu et al., 2022). For example, in the random survival forest (RSF) model, the parameters ntree (number of trees) and mtry (number of variables per split) were optimized via grid search within a leave-one-out cross-validation (LOOCV) framework. For Enet, Lasso, and Ridge, the regularization parameter λ was determined using LOOCV, and the L1–L2 balance parameter α was tuned from 0 to 1 in increments of 0.1. Similar LOOCV-based optimization strategies were applied for CoxBoost, plsRcox, SuperPC, GBM, and survival-SVM, with details including penalty factors, number of components, boosting steps, and tree counts. Feature selection was inherently performed by embedded methods such as Lasso, stepwise Cox, CoxBoost, and RSF, which identify predictive variables based on internal criteria (e.g., AIC or shrinkage penalties). All datasets were standardized using Z-score normalization prior to modeling. The TCGA dataset was used as the discovery cohort, while E-MTAB-1980, ICGC, and GSE22541 served as independent validation cohorts. Model development was carried out using 10-fold cross-validation in the discovery cohort across 101 algorithm combinations. The final MLRS model was selected based on the highest average concordance index (C-index) across the validation datasets.

### 2.4 Prognostic significance of the MLRS and development of the nomogram

Each sample in the discovery and validation datasets was scored and sorted into low- and high-MLRS groups based on the best-generating model. The prognostic significance and performance of the MLRS were evaluated through Kaplan-Meier survival curves and time-dependent receiver operating characteristic (ROC) curves, respectively. Moreover, we meticulously identified 101 published signatures developed for ccRCC for performance comparison with MLRS. The C-index was calculated to evaluate the predictive ability of all signatures in each dataset.

We then explored the correlation between the MLRS and different clinical features. A stratified survival analysis was conducted to reveal the prognostic significance of the MLRS in ccRCC patients. We performed Cox regression analyses to identify the independent predictors of outcomes for ccRCC patients. To further improve the accuracy of the MLRS in predicting patient prognosis and facilitating clinical application, we combined clinical variables with independent prognostic efficacy to establish a nomogram.

### 2.5 Functional enrichment analysis

Gene Ontology (GO) and Kyoto Encyclopedia of Genes and Genomes (KEGG) analyses were performed to explore the functions and pathways of the intersecting genes identified from the DEGs via WGCNA. Additionally, to explore the different GO terms in different MLRS groups, we carried out gene set enrichment analysis (GSEA) with the GO gene set (c5. all.v2023.2. Hs.symbols.gmt) using the “clusterProfiler” R package. Gene set variation analysis (GSVA) was further employed to analyze the variations in hallmark pathway activities (h.all.v2023.1. Hs.symbols.gmt) between the low- and high-MLRS groups through the “GSVA” R package.

### 2.6 Comprehensive analysis of tumor immune cell infiltration

Single-sample gene set enrichment analysis (ssGSEA) is commonly used to infer the enrichment scores of a particular gene set for each sample with the “GSVA” R package. In our study, we compiled several previously published signatures associated with tumor microenvironment (TME) cell types, immune suppression, and immune exclusion. Then we utilized ssGSEA to analyze the immunological differences between the two groups comprehensively. Using the CIBERSORT algorithm, we also quantified the infiltration of 22 immune cell types to explore the correlation, differences, and prognosis of TME-infiltrating cell types in ccRCC patients based on the MLRS.

### 2.7 Immunotherapy response and drug sensitivity estimation

For the immunotherapy response, we investigated the patients’ delayed response survival to immunotherapy using the IMvigor210 cohort, which included 348 urothelial carcinoma patients who were administered atezolizumab (Mariathasan et al., 2018). Tumor immune dysfunction and exclusion (TIDE, http://tide.dfci.harvard.edu/) was used to predict immunotherapy response by stimulating tumor immune evasion. In addition, we used three additional immunotherapy datasets (GSE78220, GSE135222, and GSE91061) to confirm our results.

To identify drug candidates associated with increased drug sensitivity in patients with high-MLRS, we first obtained drug sensitivity data for human cancer cell lines from the CTPR (https://portals.broadinstitude.org/ctrp) and PRISM (https://depmap.org/portal/prism/) databases. Drug sensitivity was estimated using the area under the dose–response curve (AUC), with lower AUC values indicating increased sensitivity to a specific compound. A ridge regression-based algorithm built in the “oncoPredict” R package was used to detect drug responses and compare differences in drug sensitivity between the low—and high-MLRS groups.

### 2.8 scRNA-seq data collection and analysis

Single-cell RNA sequencing (scRNA-seq) data from 7 patients with ccRCC tissues were obtained from the GSE210042 dataset (Davidson et al., 2023). The raw data were transformed into Seurat objects for downstream analysis using the “Seurat” R package. Cells with fewer than 300 genes and more than 10% mitochondrial gene count and those with fewer than three genes detected were excluded. The top 2000 highly variable genes were identified, followed by scaling and dimensionality reduction. The “Harmony” R package was then applied to the joint datasets to remove batch effects. Moreover, the results were presented by the UMAP algorithm, and typical marker genes were utilized for cell annotation. The intercellular communication between different cell subtypes in ccRCC was predicted with the “CellChat” R package with default parameters based on the analysis of ligand‒receptor interactions to model the communication probability and determine significant communications. Finally, we conducted pseudotime analyses to characterize the developmental trajectory of endothelial cells using Monocle3.

### 2.9 Cell lines

Human RCC cell lines (ACHN, 786-O, 769-P, and Caki-1) and the human renal tubular epithelial cell line (HK2) were obtained from the Cell Bank of the Chinese Academy of Sciences. All cell lines were cultured in RPMI-1640 medium (Gibco) supplemented with 10% fetal bovine serum (FBS; Gibco) and maintained at 37°C in a humidified 5% CO_2_ incubator. HUVECs were cultured in Endothelial Cell Medium (ECM; ScienCell) supplemented with 5% FBS, 1% endothelial growth supplement, and 1% penicillin-streptomycin. The EMCN overexpression plasmid and corresponding control vector were purchased from Genechem (Shanghai, China), and transfection was performed using Lipofectamine 3,000 (Invitrogen) according to the manufacturer’s instructions. For knockdown experiments, EMCN-targeting siRNAs (si-EMCN#1 and si-EMCN#2) and control siRNA (si-NC) were synthesized by GenePharma (Shanghai, China) and transfected using Lipofectamine 3,000.

For the co-culture assay, HUVECs were transfected with siRNAs for 48 h, then seeded into the upper chambers of 0.4 μm Transwell inserts (Corning). Human ccRCC cell lines (786-O or Caki-1) were seeded into the lower chambers. After 48 h of co-culture, ccRCC cell proliferation was assessed using the Cell Counting Kit-8 (CCK-8), and OD450 values were measured using a microplate reader.

### 2.10 Immunohistochemistry and real-time quantitative PCR (RT‒qPCR)

The Human Protein Atlas (HPA, https://www.proteinatlas.org/) database, a protein expression database, was employed to compare the protein expression of *EMCN* between normal and ccRCC samples via immunohistochemical staining.

Total RNA was extracted from the cells using TRIzol (Invitrogen, Carlsbad, CA, United States). The PrimeScript RT Reagent Kit (TaKaRa, Tokyo, Japan) was used for reverse transcription of total RNA to cDNA. RT-qPCR assays were carried out using the CFX96 Real-Time PCR Detection System (Bio-Rad, Hercules, CA, United States). Relative gene expression was calculated using the 2^−ΔΔCT^ method and was normalized to *GAPDH* expression. The primer sets for *EMCN* were 5′-AGCAACCAGCCGGTCTTATTC-3′ (forward) and 5′-AGCACATTCGGTACAAACCCA-3′ (reverse). The primer sets for GAPDH were 5′-ATCATCCCTGCATCCACT-3 (forward) and 5′-ATCCACGACGGACACATT-3′ (reverse).

### 2.11 Cell Counting Kit-8 (CCK-8) assay

Cell proliferation was assessed using a CCK-8 Kit (MA0218, Dalian Meilun Biotechnology Co.) according to the manufacturer’s protocol. Briefly, cells were seeded onto 96-well plates (5 × 10^3^ cells/well) and cultured for various durations (1, 2, 3, and 4 days). Then, 10 μL of CCK-8 reagent was added to each well, and the cells were incubated for 2 h. The absorbance at 450 nm was measured using a microplate reader (Molecular Devices, LLC).

### 2.12 Statistical analysis

Statistical analyses were performed using R software (v4.3.2) and GraphPad Prism software (v9.5.1). Differences between two groups were analyzed using Student’s t-test or the Wilcoxon rank-sum (Mann‒Whitney) test. Comparisons among multiple groups were performed with one-way analysis of variance (ANOVA) and the Kruskal‒Wallis test. Group differences in the clinical characteristics of the subjects were analyzed using chi-square tests. Survival curves were plotted using the Kaplan-Meier method and compared using the log-rank test. A *p* value less than 0.05 was considered to indicate statistical significance.

## 3 Results

### 3.1 Identification of the hub gene modules and key PRGs associated with ccRCC

**FIGURE 1:**
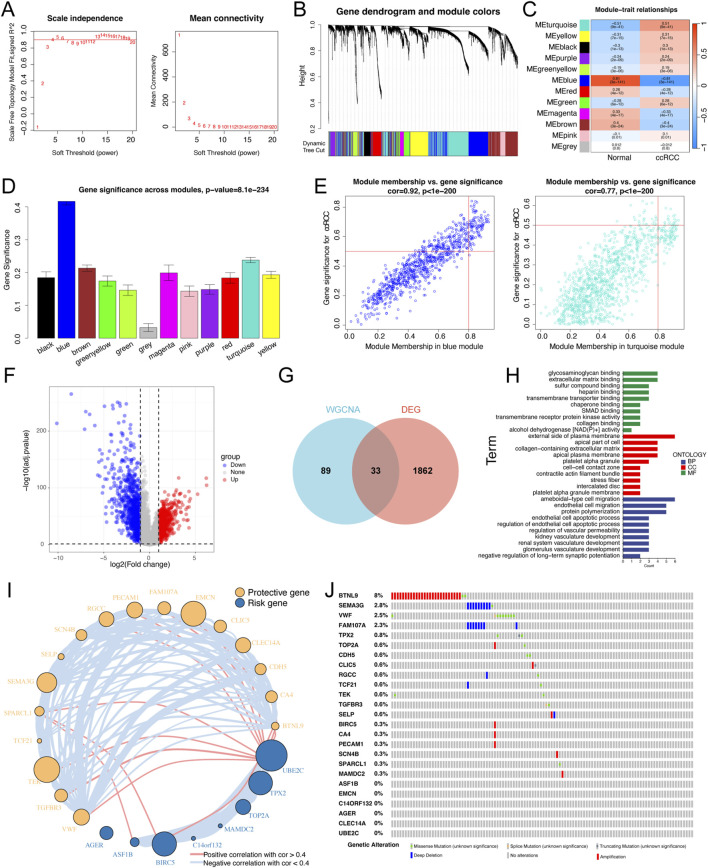
Identification of the key differential expression genes in ccRCC patients. **(A)** Analysis of the scale-free index for various soft-threshold powers (β). The left panel representing the relationship between β and scale-free R 2. The right panel representing the relationship between β and mean connectivity. **(B)** Gene dendrogram based on a dissimilarity measure (1-TOM). **(C)** A heatmap of correlation between different gene modules and clinical traits (normal vs ccRCC). **(D)** Bar plots exhibiting blue and turquoise modules are two most key modules related to ccRCC. **(E)** Scatter plots of module membership vs gene significance in the blue and turquoise modules. **(F)** Volcano plot of differential expression analysis of normal and ccRCC samples in the TCGA dataset. **(G)** Venn plot of the intersecting genes between modules (bule and turquoise) and differentially expressed genes. **(H)** GO enrichment analyses based on the key differentially expressed genes. **(I)** Univariate Cox regression analysis of key differentially expressed genes and the correlations among these genes. The circle size represented the log10 of P value obtained from univariate Cox regression analysis. **(J)** An oncoplot showing the mutation distribution of the key differentially expressed genes in ccRCC.

The workflow of this research is shown in Supplementary Figure S1. To identify hub modules significantly related to ccRCC, WGCNA was carried out using the TCGA-ccRCC dataset. The soft threshold of 4 was decided for ensuring a scale-free nature of the network (Figure 1A), and 11 gene modules were generated based on the dynamic tree cut algorithm (Figure 1B). The turquoise and blue modules exhibited the strongest negative and positive correlations with ccRCC, respectively (Figure 1C). Furthermore, the turquoise and blue modules had greater gene significance for ccRCC than did the other modules (Figure 1D). Scatterplots illustrated the linear correlation between MM and GS, with correlation coefficients of 0.77 and 0.92 for the turquoise and blue modules, respectively (Figure 1E). Ultimately, a total of 122 genes from those two modules (turquoise: 22 and blue: 12) were screened for subsequent analysis based on thresholds of GS > 0.5 and MM > 0.8.

In addition, a volcano plot further revealed 1895 DEGs between normal kidney and ccRCC tissues in the TCGA dataset, including 880 upregulated genes and 1,015 downregulated genes (Figure 1F). We ultimately screened 33 key genes by intersecting the 122 genes from the WGCNA with the DEGs (Figure 1G). GO enrichment analysis indicated that these key genes were highly enriched in biological processes (BPs), including endothelial cell migration, endothelial cell apoptotic process, and kidney vasculature development (Figure 1H). We next conducted univariate Cox regression analysis and confirmed 25 PRGs that were significantly associated with OS. To further construct and validate the MLRS, we cross-checked and found that those 25 genes were shared among all the involved bulk-seq datasets. The results of the univariate Cox regression analysis showed that out of the 25 genes, 17 were upregulated, and the remaining 8 were downregulated (Figure 1I). In addition, the interrelationships between these genes are illustrated in Figure 1I. Finally, we studied the frequency of somatic mutations, and the top ten mutations of these genes are shown in Figure 1J, with *BTNL9* having the highest mutation frequency (8%), followed by the other nine genes, ranging from 0.6% to 2.8%.

### 3.2 Construction of a prognostic MLRS using integrative machine learning

**FIGURE 2:**
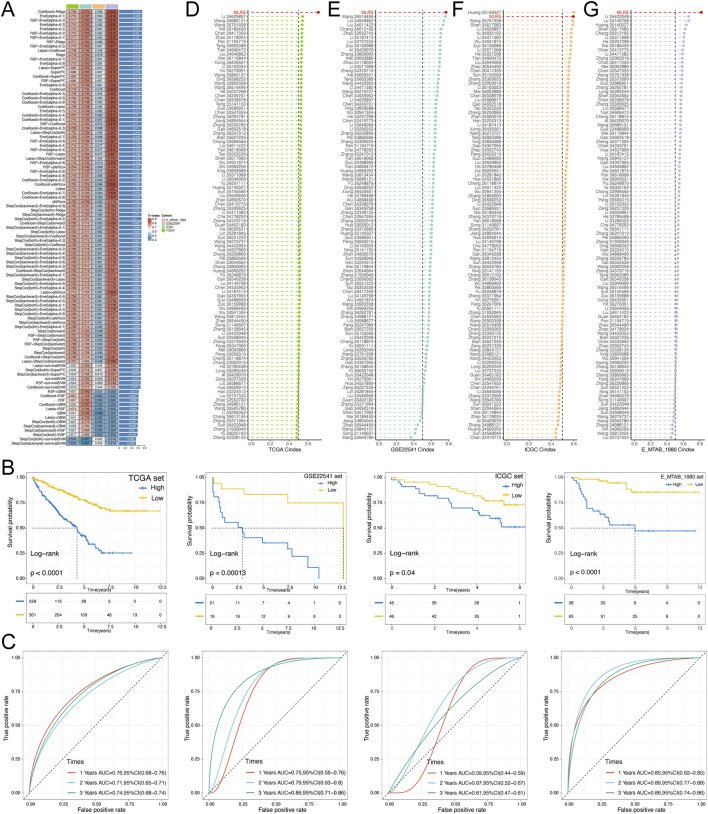
Construction and validation of a prognostic MLRS using the machine learning-based integrative procedure and comparison of the C-index among different signatures. **(A)** C-indexs of MLRS in the different (TCGA, GSE22541 and EMTAB-1980) datasets based on 101 combinations of machine learning algorithms via a 10-fold cross-validation framework. **(B)** K-M survival curves of low- and high-MLRS groups in the different datasets. **(C)** The receiver operating characteristic curve (ROC) for the performance of the MLRS in the different datasets. **(D–G)** Comparing the C-index of the MLRS and other established signatures in TCGA, GSE22541, ICGC, and EMTAB-1980 datasets.

To construct a consensus MLRS, we fitted 101 prediction models using 10 different machine learning algorithms to analyze the expression profiles of 25 prognostic genes. The TCGA-ccRCC dataset was used as the discovery cohort, whereas the E-MTAB-1980, ICGC, and GSE22541 datasets were used as the validation cohorts. Of the 101 models, the final model constructed by the combination of the CoxBoost and ridge algorithms had the highest average C-index (Figure 2A). Then, we calculated the MLRS per sample for all cohorts based on the combination of the CoxBoost and ridge algorithms, which included 8 PRGs (*BIRC5*, *TPX2*, *AGER*, *CLIC5*, *EMCN*, *MAMDC2*, *SEMA3G*, and *TEK*). Survival analysis showed that patients in the TCGA (*P* < 0.0001), E-MTAB-1980 (P = 0.00013), ICGC (P = 0.04), and GSE22541 (*P* < 0.0001) datasets suffered poor clinical outcomes in the high-MLRS group than in the low-MLRS group (Figure 2B). Moreover, the AUCs were larger than 0.7, even 0.8, suggesting that our signature had good predictive efficiency, although the AUC values did not reach such high values in the ICGC dataset (Figure 2C).

Many prognostic signatures for ccRCC have been recently published with the rapid advancement of bioinformatics technologies. To compare the prediction performance of our signature with other models, we meticulously retrieved a total of 101 published signatures involved in a variety of biological processes, such as cell death, immunity, epigenetics, and metabolism. Notably, MLRS exhibited the highest C-index in the TCGA, E-MTAB-1980, and GSE22541 datasets, except for ranking second in the ICGC dataset (Figures 2D–G). Collectively, these findings emphasized the exceptional predictive performance of the MLRS and its great potential for clinical practice.

### 3.3 Clinical relevance of the MLRS

**FIGURE 3:**
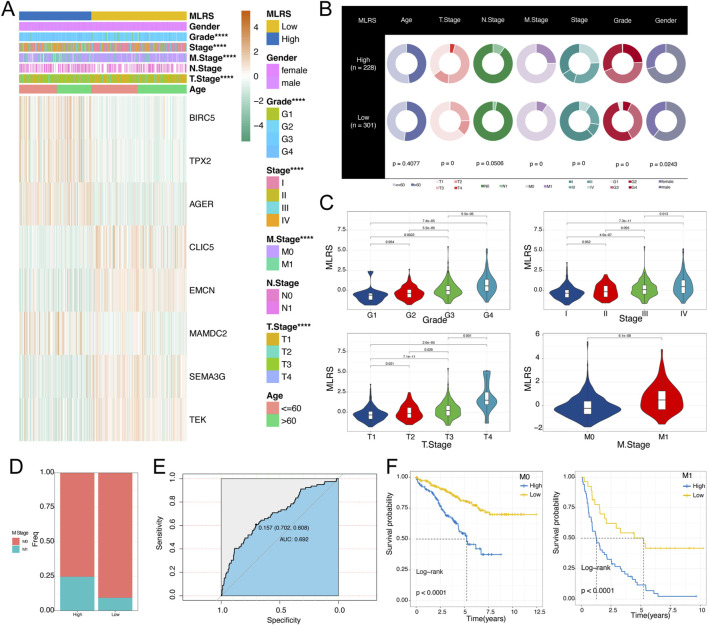
Correlation analysis between MLRS and clinical variables. **(A)** The expression profiles of the eight genes and the distribution of clinical characteristics based on the MLRS **(B)** Difference of the clinical characteristics between the low- and high-MLRS groups. **(C)** The difference in the distribution of the MLRS among ccRCC patients grouped by grade, stage, T stage, and M stage. **(D)** The proportion of M stage in the low- and high-MLRS groups. **(E)** The receiver operating characteristic curve (ROC) of MLRS in predicting the metastasis of the ccRCC. **(F)** The prognosis analysis of the MLRS for the ccRCC patients with the M stage. ***P < 0.001.

As the prognosis of ccRCC patients is commonly assessed by clinicopathological characteristics in clinical practice, we evaluated the differences in the MLRS distribution among different subgroups of clinical parameters. In the TCGA dataset, we found significant differences in the distribution of tumor grade, stage, T stage, and M stage (all *P* < 0.0001, chi-squared test) between the two groups (Figures 3A, B). Additionally, the MLRs increased as the ccRCC progressed (Figure 3C). Notably, we found that the MLRS could act as an indicator for predicting the M stage in ccRCC patients, and the MLRs was 0.692 according to the diagnostic ROC curve (Figures 3D, E), suggesting potential clinical application value of MLRS in predicting ccRCC metastases.

We then explored the ability of the MLRS to predict survival through survival analysis stratified by different clinical features. The MLRS significantly differentiated the prognosis of each clinical subgroup (including age, gender, grade, stage, T stage, N stage, and M stage) (Figure 3F; Supplementary Figure S2), with ccRCC patients in the low-MLRS subgroup having a better outcome. Moreover, we utilized the GSCA (https://guolab.wchscu.cn/GSCA/) database to comprehensively explore the multiomics phenotypes of the eight genes included in the MLRS across 33 diverse cancer types based on the TCGA dataset. The results showed that *TPX2* and *BIRC5* were upregulated, while *AGER*, *TEK*, *CLIC5*, *MAMDC2*, *EMCN*, and *SEMA3G* were mostly downregulated in multiple cancer types. Most importantly, *AGER* is a tissue-specific gene that was highly overexpressed only in ccRCC (Supplementary Figure S3A). We also noted that, except for *MAMDC2*, the other seven genes were obviously related to the prognosis (overall survival, disease-free survival, and disease-specific survival) of patients with ccRCC (Supplementary Figure S3B).

By taking into consideration the potential future clinical applications of MLRS, we screened independent prognostic parameters for MLRS by univariate and multivariate analyses (Supplementary Figure S4A) and then combined them to develop an integrated nomogram (Supplementary Figure S4B). The subsequent calibration diagram confirmed good consistency between the predicted results of the nomogram and the actual situation (Supplementary Figure S4C), and the decision curve analysis also confirmed the clinical benefit of the nomogram (Supplementary Figure S4D). In addition, the time-dependent C-index of the established nomogram had a stable and robust predictive ability and was superior to that of other clinical characteristics in predicting 1- to 10-year OS (Supplementary Figure S4E).

### 3.4 Underlying molecular mechanisms of the MLRS

**FIGURE 4:**
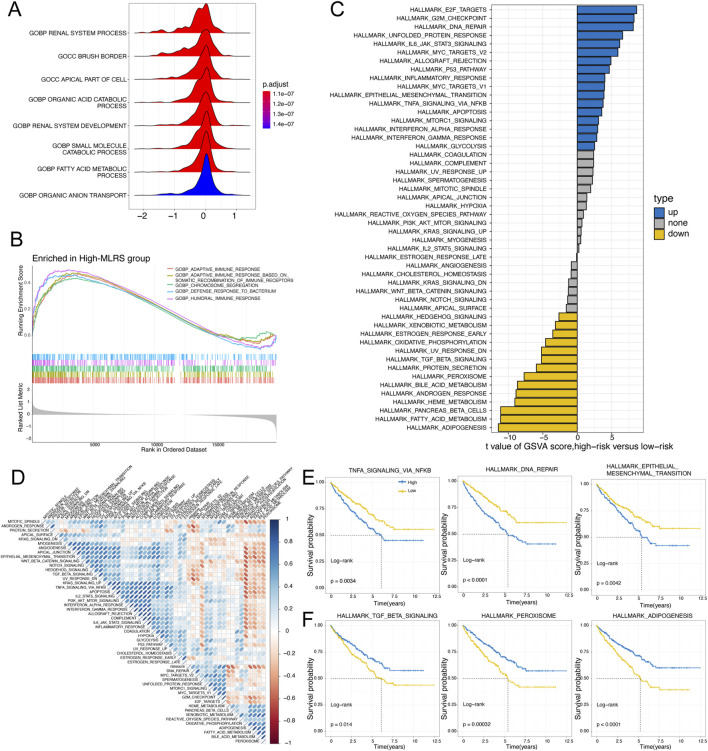
The underlying molecular features of low- and high-MLRS groups in ccRCC. **(A)** The GO terms enriched in the low-MLRS group by the gene set enrichment analysis (GSEA). **(B)** The GO terms enriched in the high-MLRS group by the GSEA. **(C)** Differences in the scores of hallmark pathway activities between the low- and high-MLRS groups by the gene set variation analysis (GSVA). **(D)** Correlation of MLRS and hallmark pathway activities scored by GSVA. **(E)** Kaplan-Meier survival curves showing the correlations between poor OS and high-MLRS group in the up-hallmark pathways. **(F)** Kaplan-Meier survival curves showing the correlations between favorable OS and high-MLRS group in the down-hallmark pathways.

To explore the underlying molecular mechanism leading to the difference in prognosis between the low- and high-MLRS subgroups, we carried out functional enrichment analysis. We employed GSEA with the GO gene set as a reference gene set and found that the low-MLRS group was enriched in renal system processes, the apical part of the cell, and several metabolic processes (Figure 4A), whereas the high-MLRS group was significantly associated with several functional terms related to immunity, such as humoral immune response, adaptive immune response, and chromosome segregation (Figure 4B). Furthermore, GSVA revealed that the low-MLRS group had a stronger correlation with pathways involved in adipogenesis, pancreatic beta cells, and other metabolic processes, while the high-MLRS group exhibited stronger activity in pathways related to E2F targets, inflammatory responses, and various cancer-related pathways, such as DNA repair, the P53 pathway, and epithelial–mesenchymal transition (Figure 4C). Correlation analysis further confirmed the unexpected associations between the MLRS and the dysregulation of multiple tumorigenic and metabolic processes in ccRCC (Figure 4D).

To elucidate whether the hallmark pathways correlated with prognosis in ccRCC patients, further analysis was conducted with Kaplan‒Meier curves. We found that pathways positively correlated with the MLRS, such as TNFA signaling via NF-κB, DNA repair, and epithelial–mesenchymal transition, were related to unfavorable prognosis (Figure 4E). Conversely, pathways negatively correlated with adipogenesis, peroxisomes, and TGF-β were related to a better prognosis (Figure 4F). These results suggested that the activation or inhibition of these pathways potentially led to the distinct outcomes observed in the different MLRS subgroups, which in part explained why the high-MLRS subgroup had a worse prognosis.

### 3.5 Relation of the tumor microenvironment to the MLRS

**FIGURE 5:**
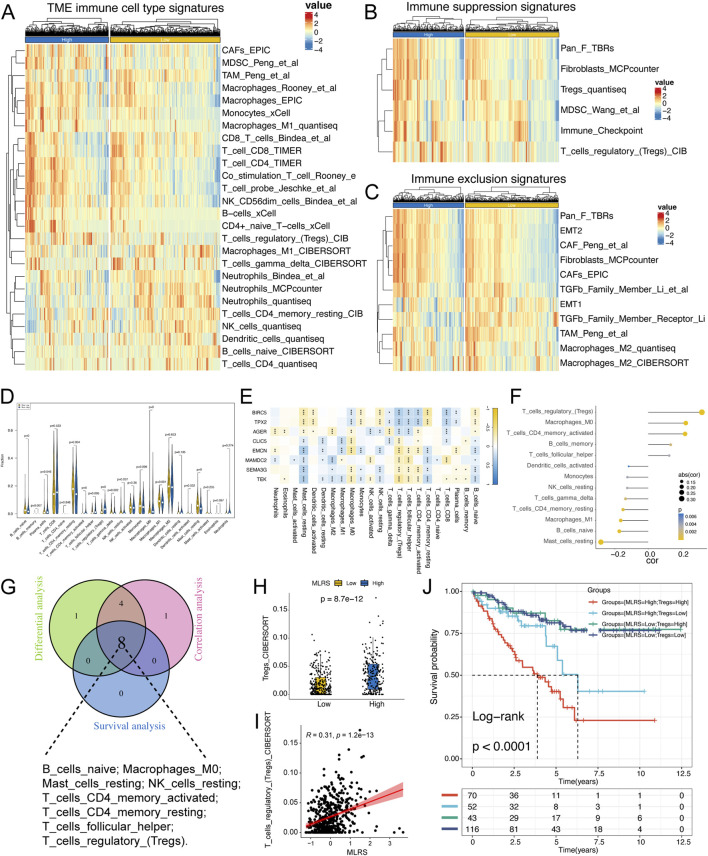
The immune landscape of low- and high-MLRS groups in ccRCC. **(A)** The distribution of tumor microenvironment (TME) immune cell type signatures between low- and high-MLRS groups. **(B)** The distribution of immune suppression signatures between low- and high-MLRS groups. **(C)** The distribution of immune exclusion signatures between low- and high-MLRS groups. **(D)** The abundance of each TME-infiltrated cell types quantified by the CIBESORT algorithm between low- and high-MLRS groups. **(E)** The correlations between TME-infiltrated cell types and eight genes built-in MLRS. **(F)** The correlations between TME-infiltrated cell types and MLRS. **(G)** Venn diagram determining the intersecting TME-infiltrated cell types among the differential, correlation, and survival analyses. **(H)** The distribution of Tregs between low- and high-MLRS groups. **(I)** The correlations between MLRS and Tregs. **(J)** Survival analysis combined MLRS with Tregs. *P < 0.05, **P < 0.01, ***P < 0.001.

To characterize the immunologic landscape of ccRCC patients, we performed a comprehensive analysis of TME-related pathway scores using the ssGSEA algorithm and found that the infiltration levels of immune cells, such as CD4^+^ T cells, CD8^+^ T cells (TIMER method), B cells (CIBERSORT method), and M1 macrophages (Quantieq method), were significantly greater in patients with high MLRS than in those with low MLRS (Figure 5A), indicating immune activation status. We also discovered that some immunosuppression- and exclusion-related pathways, such as those involving Tregs, fibroblasts, TGF- family members, and immune checkpoints were significantly enriched in the high-MLRS group (Figures 5B, C), suggesting that the ccRCC patients in the high-MLRS group might have a greater immunosuppression degree. These findings strongly implied that ccRCC patients with low MLRS values were significantly more inclined to be reclassified as having “hot tumors”, whereas those with high MLRS values were more likely to have “cold tumors”.

Using the CIBERSORT algorithm, we systematically evaluated the infiltration scores of 22 types of immune cells in ccRCC and compared the differences between the low- and high-MLRS groups. We found that naïve B cells, memory B cells, resting memory CD4 T cells, gamma delta T cells, M1 macrophages, activated DCs, and resting mast cells were more enriched in the low-MLRS group, while plasma cells, activated memory CD4 T cells, follicular T cells, regulatory T cells, resting NK cells, and M0 macrophages were obviously associated with the high-MLRS group (Figure 5D). Furthermore, we observed that eight genes in the MLR were significantly correlated with tumor immune infiltrates, of which three upregulated genes (*BIRC5*, *TPX2*, and *AGER*) in ccRCC were positively correlated with CD8^+^ T cells, and the remaining five genes showed a negative correlation with Tregs, consistent with their prognostic results in ccRCC (Figure 5E).

We then performed Spearman’s correlation analysis and identified 13 immune cells whose infiltration levels were significantly associated with the MLRS (*P* < 0.05) (Figure 5F). We also used Kaplan‒Meier curves to explore the effect of the enrichment status of immune-infiltrating cells on the prognosis of ccRCC patients. The results revealed that 8 TME-infiltrating cell types were significantly associated with OS (log-rank test, *P* < 0.05), suggesting that immune cell infiltration in ccRCC tissues might play an important role in tumor prognosis (Supplementary Figure S5). We ultimately identified eight crossed immune cell types by integrating the differential expression analysis, correlation analysis, and survival analysis (Figure 5G). Tregs may be effective targets for cancer immunotherapy; therefore, we explored the difference in Treg content between the two groups and found that the low-MLRS group had lower enrichment of Tregs, suggesting that the low-MLRS group may undergo immune-mediated tumor clearance (Figures 5H, I). We further performed survival analysis and found that the MLRS could be used as an effective complementary factor for Tregs to determine the prognosis of ccRCC patients (Figure 5J).

### 3.6 MLRS correlate with immunotherapy response and drug sensitivity

**FIGURE 6:**
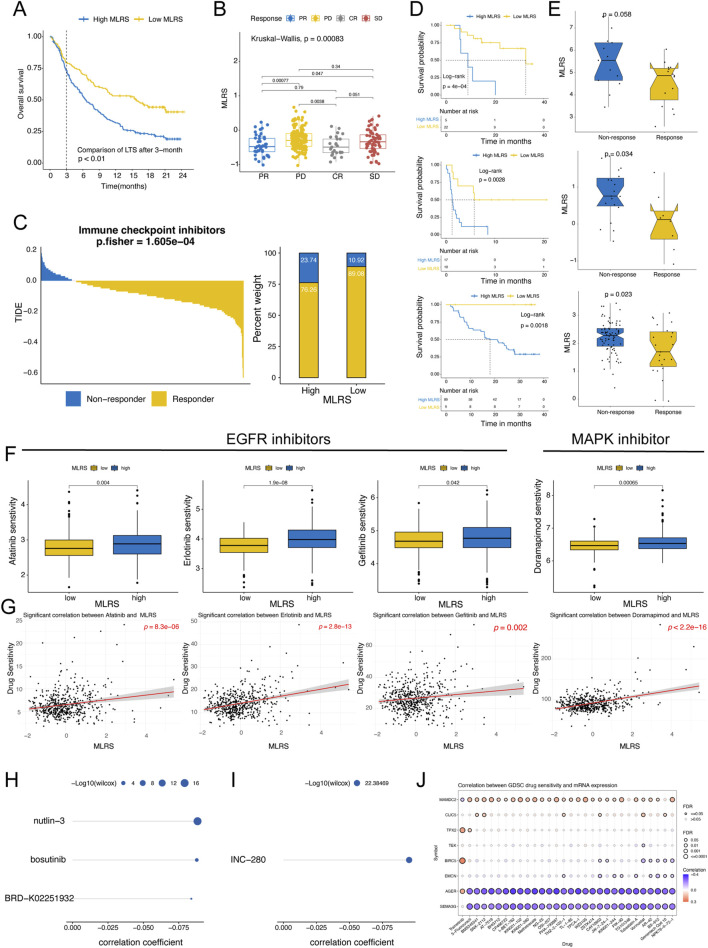
Predictive value of the MLRS in immunotherapy response and drug sensitivity. **(A)** The long-term survival (LTS) difference after 3 months of treatment between low- and high-MLRS groups in the IMvigor210 dataset. **(B)** The distribution of MLRS in patients with different immunotherapy responses in the IMvigor210 dataset. **(C)** The tumor immune dysfunction and exclusion (TIDE) algorithm predicting the immunotherapy response between low- and high-MLRS groups. **(D)** Survival analysis of low- and high-MLRS groups in the GSE78220, GSE135222, and GSE91061 datasets. **(E)** The distribution of MLRS in patients with different immunotherapy responses in the GSE78220, GSE135222, and GSE91061 datasets. **(F)** A comparison of the sensitivity to EGFR and MAPK inhibitors, including afatinib, erlotinib, gefitinib, and doramapimod between low- and high-MLRS groups. **(G)** The correlations between drug sensitivity of afatinib, erlotinib, gefitinib, and doramapimod and MLRS. **(H, I)** The AUC values of compounds from CTRP **(H)** and PRISM **(I)** databases were estimated based on the TCGA sample dataset, and Spearman correlation analysis was conducted on MLRS and AUC values. **(J)** The correlations between drug sensitivity and eight genes built-in MLRS.

To comprehensively evaluate the performance of the MLRS for predicting immunotherapeutic efficacy in ccRCC patients, we first conducted a detailed analysis of urogenital cancer patients treated with PD-L1 blockade in the IMvigor210 cohort. Unlike previous analytical approaches, we compared differences in long-term survival among patients with ccRCC after 3 months of treatment more concretely by considering the delayed clinical effects of ccRCC immunotherapy. The results indicated that patients in the low-MLRS group had a better prognosis, suggesting that they may benefit more from immunotherapy (Figure 6A). The distribution of MLRS also showed that the MLRS was significantly higher in the non-responders (progressive disease [PD] and stable disease [SD]) than in the responders (complete response [CR] and partial response [PR]) in the IMigor210 cohort (Figure 6B). Furthermore, the TIDE algorithm also displayed a better immunotherapy response in the low- MLRS group (*P* < 0.001; Figure 6C). Finally, we investigated the prognostic value of the MLR in the three independent immunotherapy cohorts separately. Patients with low MLRS tended to have significantly better prognostic outcomes than those with high MLRS in the GSE78220 (*P* = 0.0013), GSE135222 (*P* = 0.00047), and GSE91061 (*P* = 0.00018) datasets (Figure 6D). Although there was no visible difference between the low- and high-MLRS groups, a low MLR tended to be associated with greater clinical benefit in the GSE78220 cohort (Wilcox test, *P* = 0.058, Figure 6E). In addition, patients with a low MLRS may benefit more from immunotherapy in the GSE135222 (Wilcox test, *P* = 0.034) and GSE91061 (Wilcox test, *P* = 0.023) datasets.

Given that chemotherapeutic agents have significant therapeutic implications for cancers, we used multiple drug response databases to identify therapeutic compounds that were strongly associated with the MLRS in ccRCC patients. First, we examined the sensitivity of the low- and high-MLRS groups to chemotherapeutic drugs using “oncoPredict” package. The findings indicate that the sensitivities to EGFR inhibitors (afatinib, erlotinib, and gefitinib) and MAPK inhibitors (doramapimod) were significantly greater in the high-MLRS group (Figure 6F), indicating the potential importance of these agents in inhibiting the progression of malignant tumors. We also found that the MLRS was positively correlated with drug sensitivity (Figure 6G). Subsequently, we calculated the AUC values of the compounds from CTRP and PRISM and performed Spearman correlation analysis on the MLRS and AUC values based on the TCGA sample dataset. An increasing AUC represents decreased drug sensitivity. A total of four compounds (CTRP: nutlin-3, bosutinib, and BRD−K02251932; PRISM: INC−280) were found to be negatively correlated with the MLRS, suggesting potential sensitivity in the high-MLRS group (Figures 6H, I). Finally, we found that the expression of *AGER* and *SEMA3G* was strongly negatively correlated with multiple drug sensitivities, whereas *MAMDC2* showed the opposite trend (Figure 6J).

### 3.7 scRNA-seq transcriptome data and communication network of ccRCC

**FIGURE 7:**
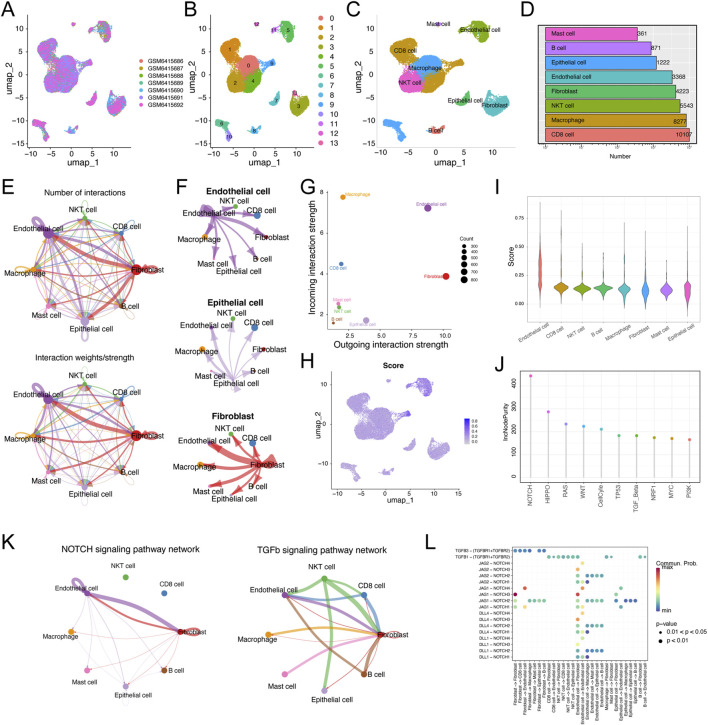
Single-cell RNA transcriptome data analysis in ccRCC. **(A)** Uniform manifold approximation and projection (UMAP) showing the integration of seven samples in the GSE210042 **(B)** UMAP visualization of 13 separate cell clusters. **(C)** UMAP of representative eight major cell types and distribution annotations in ccRCC. **(D)** Number of representative eight major cell types. **(E)** Cell-cell communications of major cell types by CellChat analysis based on interaction numbers and strength. **(F)** Network plot displaying the signaling sent from endothelial cells, epithelial cells, and fibroblasts. **(G)** Visualization of dominant senders (sources) and receivers (targets) in a scatter plot. **(H)** The activity score of MLRS in major cell types. **(I)** The distribution of the MLRS in different cell types. **(J)** The single sample gene set enrichment analysis (ssGSEA) and random forest algorithms jointly illustrating that NOTCH is the most important hallmark of cancer among the endothelial cells. **(K)** NOTCH and TGF-β signaling are the main signaling pathways involved in the interaction between endothelial cells and other cells. **(L)** The main ligand-receptor paired in NOTCH and FTG-b signaling pathways among major cell types.

Next, we investigated the detailed distribution of MLRS in ccRCC patients via a scRNA-seq dataset (GSE210042). After filtering with strict standards, 30,663 single cells from seven ccRCC samples were screened for further analysis (Figure 7A). We conducted PCA and UMAP downscaling analysis using the top 2,000 highly variable genes and obtained 13 different cell clusters (Figure 7B). Based on the expression of well-recognized marker genes previously reported, all cells were categorized into eight cell types, namely, CD8^+^ T cells, macrophages, NKT cells, fibroblasts, endothelial cells, epithelial cells, B cells, and mast cells (Figure 7C). The cell numbers of the eight cell types are shown in Figure 7D. To elucidate how these cells regulate tumorigenesis, we utilized CellChat to construct cell communication networks to comprehensively evaluate the potential molecular interactions between ligand‒receptor pairs and major cell types. We investigated the number and weight/strength of interactions between diverse cell types and found that endothelial cells play a considerable role in intercellular communication (Figure 7E). In addition, fibroblasts and epithelial cells communicate closely with endothelial cells, and the signals sent from these cells are shown in Figure 7F. The outgoing and incoming interaction strengths of the cells also indicated that fibroblasts and endothelial cells emitted more signals, and that macrophages and endothelial cells received more signals than other cells (Figure 7G). The enrichment scores of MLRS were estimated by “AUCell”, and the results clearly showed that MLRS were highly enriched in endothelial cells (ECs) compared to other cell types (Figures 7H, I).

To further address the role of endothelial cells in ccRCC progression, we explored the significance of the hallmark of cancer and found that NOTCH was considered the most important of the various cancer hallmarks (Figure 7J). Notably, the interactions between every gene in the gene set and the NOTCH signaling pathway further highlighted a significant positive correlation between five genes (*TEK*, *EMCN*, *CLIC5*, *SEMA3G*, *AGER*) and the NOTCH pathway (Supplementary Figure S6). Combined with the results of the CellChat and random forest algorithm analyses, the NOTCH and FTG-b signaling pathways were identified as the main signaling pathways involved in the interaction between endothelial cells and other cells (Figure 7K). The expression levels of ligands and receptors in the NOTCH and FTG-b signaling pathways are shown in Figure 7L. The communication between epithelial cells and other cell types involves specific ligand‒receptor pairs, such as JAG1-NOTCH3 and TGFB1-(TGFBR1+TGFBR2).

### 3.8 *EMCN* correlates with the differentiation fate of endothelial cells in ccRCC

**FIGURE 8:**
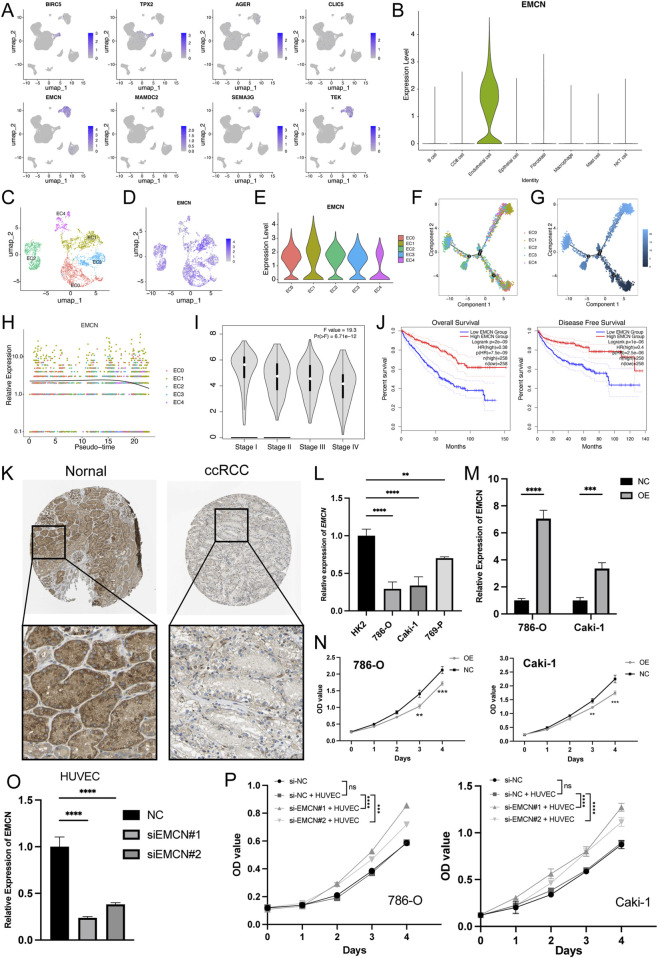
*EMCN* is specifically expressed in endothelial cells and inhibits the ccRCC cell proliferation. **(A)** Uniform manifold approximation and projection (UMAP) of eight genes expression built-in MLRS. The color shade showing the expression of the corresponding gene. **(B)** Violin plots visualizing the normalized expression levels of *EMCN* across the major cell types. **(C)** UMAP showing the five subclusters (EC0-5) of the endothelial cells. **(D)** The expression of *EMCN* in five different subclusters of endothelial cells with different color degrees. **(E)** Violin plots visualizing the normalized expression levels of *EMCN* across the five different subclusters of endothelial cells. **(F)** The pseudotime trajectory describing the distribution of five different subclusters of endothelial cells. **(G)** Differentiation trajectory of endothelial cells in ccRCC, color-coded for pseudotime. **(H)** In the dynamic expression profile of *EMCN* in endothelial cells trajectory. **(I)** Distribution of *EMCN* expression in ccRCC at different stages. **(J)** Kaplan Meier curve analysis for the OS and PFS based on the *EMCN* expression in ccRCC. **(K)** Distribution of protein expression in normal renal and ccRCC tissues obtained from the Human Protein Atlas (HPA) database. **(L)** *EMCN* expression levels in different ccRCC cell lines and immortalized proximal tubule epithelial cells (HK2). **(M)** qRT-PCR results confirming the *EMCN* was successfully overexpressed in 786-O and Caki-1 cells. **(N)** CCK-8 assay showing that overexpression of *EMCN* obviously inhibited the proliferation of 786-O and Caki-1 cells. **(O)** Quantitative RT-PCR analysis confirmed the knockdown efficiency of *EMCN* in HUVECs transfected with si-EMCN#1 and si-EMCN#2 compared to control siRNA (si-NC). **(P)** CCK-8 assays showing the proliferation of 786-O and Caki-1 cells after 4-day co-culture with HUVECs transfected with si-NC, si-EMCN#1, or si-EMCN#2 in a Transwell system.**P < 0.01, ***P < 0.001, ****P < 0.0001.

Subsequently, we analyzed the expression profiles of eight genes in the MLRS across the different cell types and found that *EMCN* was specifically expressed in endothelial cells (Figures 8A, B). Endothelial cells were further subdivided into five clusters (EC0-4) after UMAP dimensionality reduction (Figure 8C), and the expression of *EMCN* among EC subclusters is displayed with different degrees of color (Figure 8D). Notably, EC1 and EC4 exhibited the highest and lowest expression of *EMCN*, respectively (Figure 8E). Next, noted that the progression trajectory originated from the EC0 and clustered into different branches using the pseudotime trajectory analysis. We also performed a pseudotime trajectory analysis of endothelial cells to dissect the evolutionary dynamics of ccRCC endothelial lineages (Figure 8F). The evolutionary trajectory of endothelial cells originated from EC0 and developed into different branches, with EC1 and EC4 cells in the upper right corner and EC2 and eEC3 cells on the left (Figure 8G). Pseudotemporal ordering demonstrated that the *EMCN* decreased in the latter stages of differentiation (Figure 8H).

Furthermore, *EMCN* expression correlated with pathological grade and decreased stepwise and significantly decreased as the ccRCC progressed (Figure 8I). Survival curves further showed that patients with high *EMCN* expression exhibited better clinical outcomes, indicating that the loss of endothelial *EMCNs* might drive ccRCC growth (Figure 8J). Consistent with our earlier observations, representative immunohistochemical staining results showed that the level of *EMCN* expression was significantly lower in ccRCC tissues than in normal tissues (Figure 8K). *EMCN* mRNA expression was markedly lower in ccRCC cell lines (786-O, Caki-1, and 769-P) than in HK2 (Figure 8L). To confirm the role of *EMCN* in ccRCC development, we altered its expression by transfecting 786-O and Caki-1 cells with *EMCN* overexpression plasmids, and the results were confirmed by qRT‒PCR analysis (Figure 8M). The CCK-8 assay results revealed that overexpression of *EMCN* inhibited the proliferation of 786-O and Caki-1 cells (Figure 8N), confirming the role of *EMCN* as a tumor suppressor in ccRCC. To further investigate whether endothelial-derived *EMCN* contributes to tumor regulation, HUVECs were transfected with si-*EMCN* or si-NC (Figure 8O) and subsequently co-cultured with 786-O and Caki-1 cells using a non-contact Transwell system. After 48 h of co-culture, CCK-8 assays were performed on ccRCC cells. The results showed that ccRCC cells co-cultured with *EMCN*-silenced HUVECs exhibited significantly enhanced proliferation compared to the control group (Figure 8P), indicating that *EMCN* knockdown in endothelial cells may promote tumor growth by modulating the tumor microenvironment.

## 4 Discussion

CcRCC is the predominant solid tumour of the kidney and the most fatal among all urological cancers (Sung et al., 2021). A significant number of patients have exhibited a limited response to immunotherapeutic treatment, despite the presence of PD-1 in ccRCC patients (Au et al., 2021). Consequently, dependable biomarkers are critically required to assess the prognosis of ccRCC patients and to categorise those who may derive advantages from immunotherapy.

To establish a prognostic signature with consensus efficacy for ccRCC patient prognosis and enhance its clinical application, we employed 10 machine learning techniques to develop a streamlined translational model (Liu et al., 2022). We calculated the average C-index of the four included datasets as the ranking criterion to mitigate the risk of overfitting and biassed outcomes resulting from the optimisation of the training cohort. Our findings indicated that the application of the CoxBoost and ridge algorithms yielded an exceptional C-index in the discovery (TCGA) dataset as well as in three validation datasets (GSE22541, E-MTAB-1980, and ICGC). However, it is difficult to generalize the results to the ICGC dataset, likely due to this dataset focused on RCC but was not limited to clear cell subtypes. RCC encompasses several subtypes, including clear cell, papillary, and chromophobe renal cancers, which exhibit significant genetic and clinical heterogeneity. This heterogeneity among subtypes may contribute to the decreased predictive accuracy of our model in the ICGC dataset (Rose and Kim, 2024). Furthermore, technical differences in sample processing and sequencing platforms across datasets might also influence the results. Notably, MLRS exhibited better or at least comparable prediction performance in each cohort compared with other published studies. A nomogram combining MLRS with multiple clinical features could further improve the predictive ability of the nomogram in comparison to that of a single feature.

The prognostic MLRS was constructed using the eight most valuable genes, five of which have been documented as contributors to the course of ccRCC. The expression of *BIRC5*, or survivin, was an independent predictor of ccRCC advancement and adversely correlated with patient survival, consistent with our data (Parker et al., 2006). *TPX2*, a regulator of Aurora-A, was established as correlated with advanced ccRCC grade and stage and identified as an independent predictor of recurrence in a tissue microarray comprising 207 patients (Glaser et al., 2017). Importantly, the signaling cross-talk between *TPX2* and Aurora B appears to be essential for the accurate completion of mitosis. The latter can interact with *BIRC5* to play a critical role in regulating mitotic spindle assembly and chromosome segregation, both of which are crucial for cell division (Iyer and Tsai, 2012). *AGER*, the receptor for advanced glycation end products, plays a crucial role in tumor angiogenesis and can be used as a prognostic biomarker for ccRCC (Guo et al., 2015). The expression of *EMCN* (also called endomucin), which is expressed in endothelial cells, was downregulated and associated with better overall survival in VHL-mutant ccRCC patients (Wang et al., 2022). The role of AGER role in tumor angiogenesis could be influencing EMCN expression in endothelial cells, thereby affecting tumor microenvironment and vascularization, which is critical for tumor growth and metastasis (Swanner et al., 2023). A novel podocyte gene, *SEMA3G* (semaphorin 3G) might contribute to ccRCC tumorigenesis by affecting Wnt pathway (Wang et al., 2023). Other reports have suggseted that *TEK* knockdown not only functionally promoted cell proliferation and migration but also affected cell apoptosis by regulating AKT phosphorylation (Chen et al., 2021). Recently, two other genes (*CLIC5* and *MAMDC2*) have also been reported to function as tumor suppressors in some cancers (Lee et al., 2020; Chen et al., 2021). Despite limited data on ccRCC.

The types and phenotypes of tumor-infiltrating immune cells in the TME are closely associated with the progression of ccRCC (Mier, 2019). Although different algorithms had different effects on immune cell infiltration, we found that ccRCC patients with high MLRs presented abundant immunosuppression- and exclusion-related pathways, such as those involving Tregs, CAFs, MDSCs, fibroblasts, TGF-family members, and immune checkpoints. Specifically,TGF-β not only helps in the conversion of naïve T cells into regulatory T cells but also enhances the fibrotic barrier around tumors, which impedes the infiltration of effector immune cells. Moreover, TGF-β interacts synergistically with other components of the tumor microenvironment to enhance immune evasion through the upregulation of PD-L1 on tumor and immune cells, thus contributing to an immunosuppressive milieu (Mariathasan et al., 2018; Batlle and Massagué, 2019). The concept of “cold” and “hot” tumor phenotypes is pivotal to the effectiveness of immunotherapy. Our findings indicate a significant correlation between the MLRS and the expression of crucial immune checkpoint molecules, which are key facilitators of immune evasion within the tumor microenvironment. These molecules are instrumental in sustaining the ‘cold’ phenotype, where tumors exhibit minimal immune cell infiltration and are less responsive to immunotherapy (Topalian et al., 2015). Elevated MLRS values might suggest an enhancement of these immune checkpoints, thereby fostering immune suppression and perpetuating a “cold” tumor environment. In contrast, ccRCC patients in the low-risk group had a highly infiltrated immune microenvironment characterized by the presence of CD4^+^ T cells, CD8^+^ T cells (TIMER method), B cells (CIBERSORT method), and M1 macrophages (Quantieq method), revealing that the low-MLRS group has stronger antitumor immune activity.

To address the poor response to immunotherapy in the high-MLRS group, implemented a thorough search technique proven helpful in identifying potentially suitable therapeutic molecules in prior research. Patients with high-MLRS exhibited heightened sensitivity to afatinib, erlotinib, gefitinib, and doramapimod. Small molecule inhibitors with specific targeting activity, which can kill tumor cells but rarely threaten normal tissues and cells, play a key role in cancer therapy (Fang et al., 2022). Thus, we predicted three CTRP-derived agents (nutlin-3, bosutinib, and BRD−K02251932) and one PRISM-derived agent (INC−280) for the treatment of high-risk patients. For example, Nutlin-3 selectively enhances cancer cell apoptosis by activating the p53 pathway (Yee-Lin et al., 2018). Notably, Nutlin-3 is nongenotoxic and protects kidney cells from the cytotoxic effect of cisplatin (Jiang et al., 2007), demonstrating its potent role in the treatment of ccRCC.

In our scRNA-seq analysis of ccRCC, we characterized cellular heterogeneity and assessed the expression patterns of MLRS genes across different cell types. While we observed that MLRS genes were more highly enriched in endothelial cells compared to other cell types, we acknowledge that this does not necessarily imply exclusivity. In agreement with other studies (Howard et al., 2013; Fendler et al., 2020), our cell communication analysis revealed that the Notch signaling pathway acted as the strongest mediator in endothelial cells. More importantly, we identified DLL4/Notch and JAG/Notch signaling as the key ligand-receptor pairs involved in ligand‒receptor interactions in ccRCC, which might disrupt angiogenesis to reduce tumor growth and metastasis (Kangsamaksin et al., 2015). We then verified the prominent role of the *EMCN* in MLRS and found that the *EMCN* correlated with the differentiation fate of endothelial cells in ccRCC. Finally, *in vitro* experiment further revealed the suppressive function of *EMCN* in regulating the proliferation of ccRCC cells. Other studies have confirmed that overexpression of *EMCN* can inhibit neutrophil adhesion *in vitro* and reduce the infiltration of CD45^+^ and NIMP-R14+ cells *in vivo* (Zahr et al., 2016). Collectively, *EMCN* has enormous potential as a new immunotherapy target and has great potential as a new therapeutic target in the progression of ccRCC.

Several limitations of our study merit attention. Firstly, the retrospective design introduces a degree of internal bias due to reliance on previously collected data, potentially affecting the generalizability of our findings. Secondly, the small sample size in our single-cell datasets restricts the scope of data analysis, necessitating caution in extending our results more broadly. Thirdly, to enhance the validity and applicability of the MLRS and to fully assess its clinical significance, a large-scale, multicenter prospective cohort study is essential. Lastly, although our study underscores the specific roles of *EMCN* in ccRCC, the underlying mechanisms through which *EMCN* impacts the immune microenvironment, along with other key genes in the MLRS, necessitate further detailed investigation.

## 5 Conclusion

Our study shows that MLRS can effectively predict the prognosis of ccRCC and is closely related to cancer progression and the immune microenvironment, offering new insights into immunotherapy response and novel strategies for ccRCC treatment.

## Data Availability

The original contributions presented in the study are included in the article/Supplementary Material, further inquiries can be directed to the corresponding author.
