# Supplementary material for: Identifying a prognostic signature for clear cell renal cell carcinoma: the convergence of single-cell and bulk sequencing with machine learning
Source: Front Cell Dev Biol. 2025 Jun 4;13:1560095. doi: 10.3389/fcell.2025.1560095 (PMC12174135; doi:10.3389/fcell.2025.1560095)
Supplement: Supplementary file 1 [file DataSheet1.docx]

**
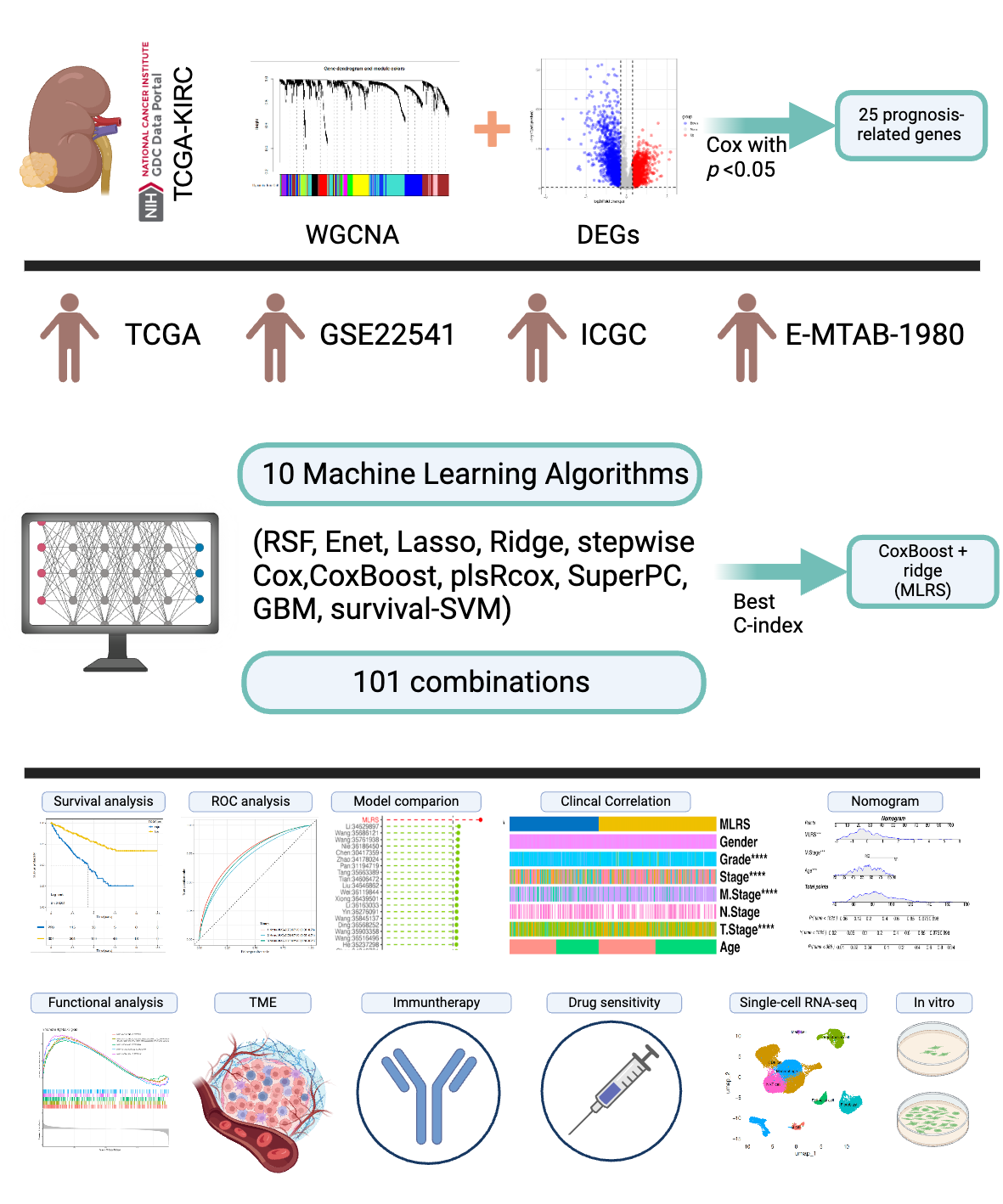
Fig. S1. The computational framework for establishing the MLRS in ccRCC.**

**
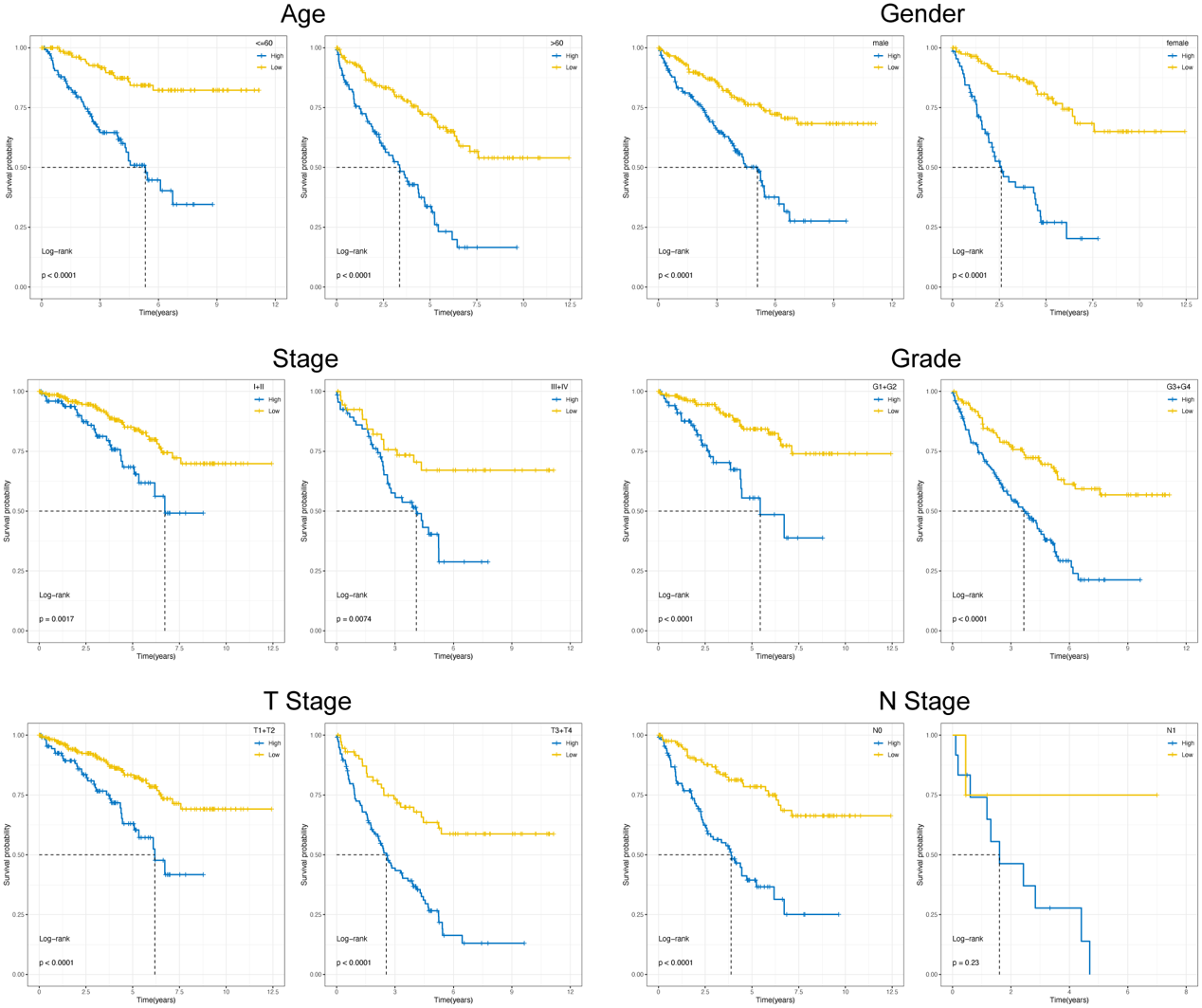
**

**Fig. S2. Kaplan-Meier survival analysis of ccRCC in low- and high-MLRS groups stratified by different clinicopathological features, including age, gender, and grade, stag, T stage, and N stage.**

**
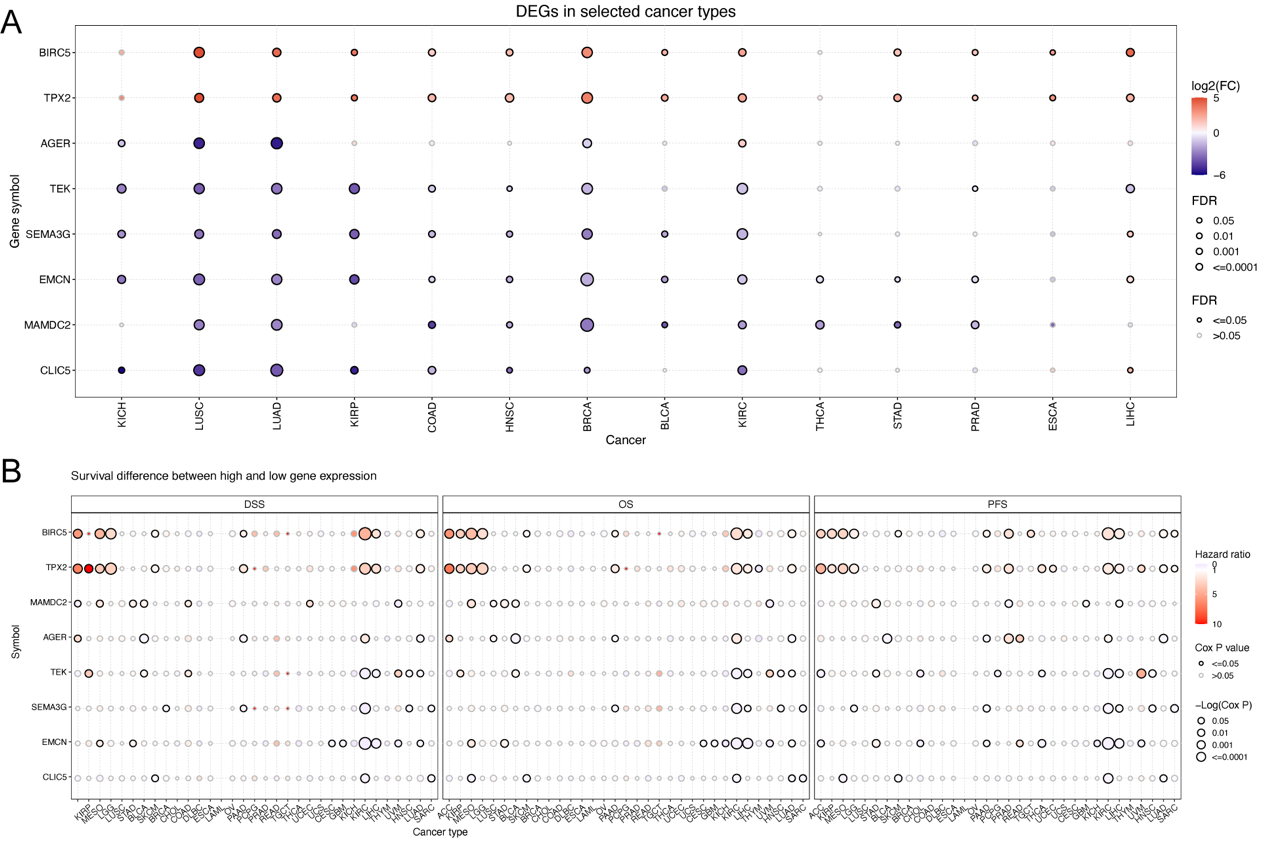
 Fig. S3. Differential expression analysis and prognostic analysis of eight genes built-in MLRS.** (A) Differential expression analysis of eight genes built-in MLRS in pan-cancer. (B) The prognostic values of eight genes built-in MLRS in pan-cancer.

**
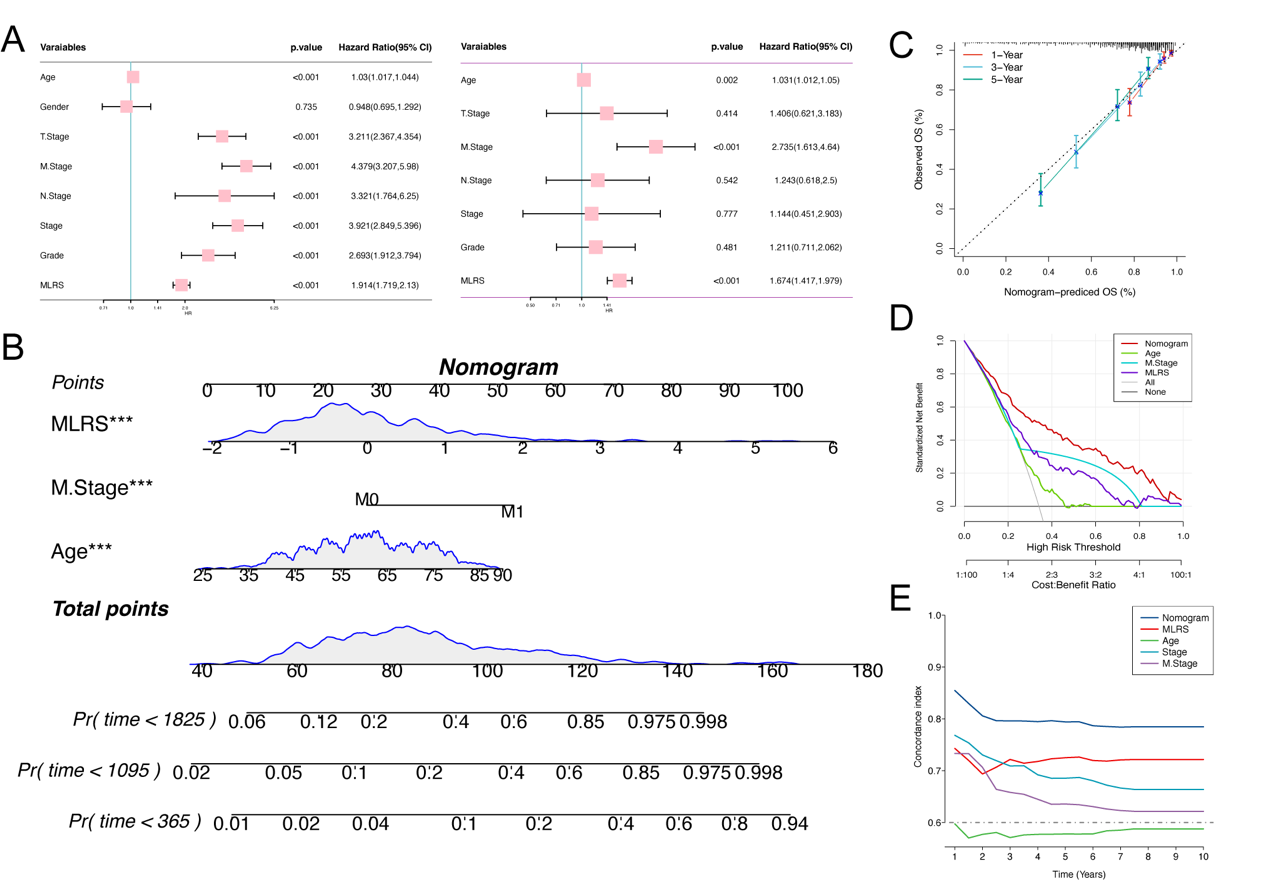
 Fig. S4. Development and validation of the nomogram.** (A) Univariate and multivariate analyses of the clinical characteristics and MLRS for the OS in the TCGA dataset. (B) Development of the nomogram based on three independent prognostic characteristics, including MLRS, age, and M stage. (C) Calibration curves of nomogram for predicting OS at 1-, 3-, and 5-year. (D) The decision curve analysis of the nomograms compared for 5-year OS. E The comparison of the C-index between the nomogram and other independent clinical characteristics.

**
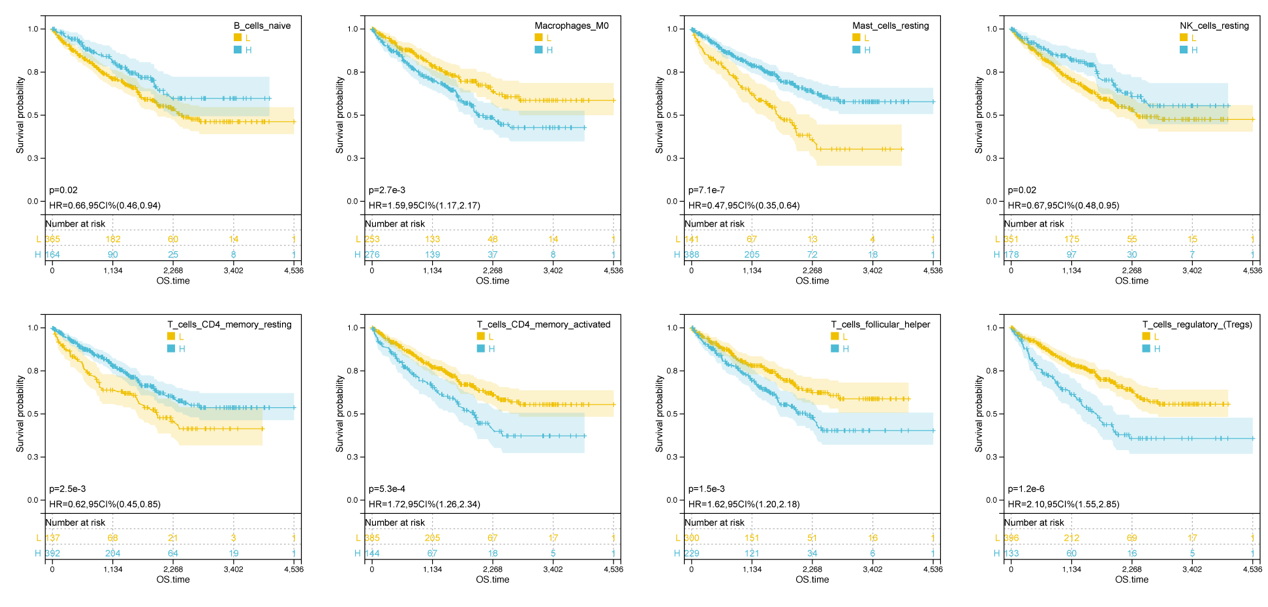
 Fig. S5. Kaplan-Meier survival analysis of 12 TME-infiltrating cell types in low- and high-MLRS groups.**


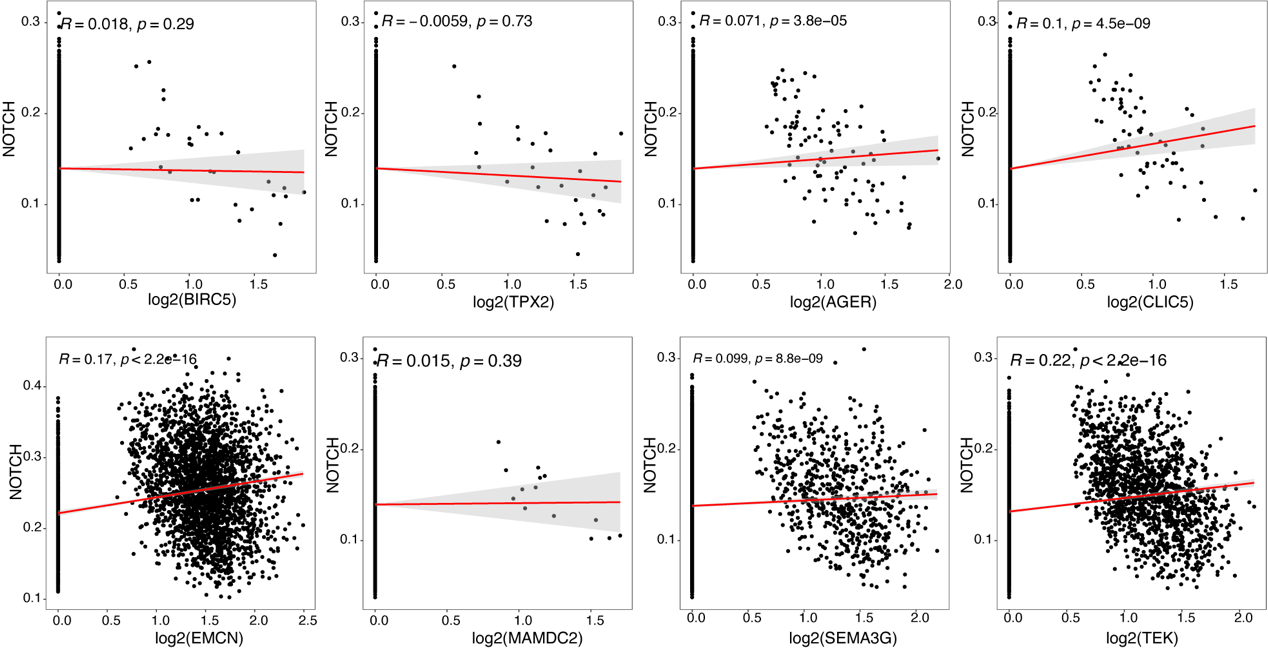


**Fig. S6. The correlation of eight MLRS genes and the NOTCH pathway.**
